# IGFBP2 Knockdown Ameliorates Diabetic Gastroparesis by Inhibiting VDAC1-Dependent Mitophagy in Interstitial Cells of Cajal

**DOI:** 10.2174/0118715303448109260613173507

**Published:** 2026-07-27

**Authors:** Xiao Yuan, Yuting Chen, Zhengran Qin, Hong Zhang

**Affiliations:** 1 Department of Endocrinology, The First Affiliated Hospital of Zhejiang Chinese Medical University (Zhejiang Provincial Hospital of Chinese Medicine), Hangzhou, China

**Keywords:** Diabetic gastroparesis, insulin-like growth factor binding protein 2, interstitial cells of cajal, mitophagy, voltage-dependent anion channel 1, mitochondrial dysfunction

## Abstract

**Introduction/Objective:**

Although insulin-like growth factor binding protein 2 (IGFBP2) is significantly upregulated in diabetic gastroparesis (DGP) and may regulate interstitial cells of Cajal (ICC) mitophagy through its interaction with voltage-dependent anion channel 1 (VDAC1), the precise mechanism of action remains unclear. This study aims to investigate the role and mechanism of IGFBP2 in the pathogenesis of DGP, with a specific focus on its impact on mitochondrial quality control in ICCs.

**Methods:**

An *in vivo* DGP model was established. IGFBP2 expression was knocked down to assess its effects on systemic metabolism (blood glucose, HbA1c), gastric motor function (gastric emptying, motility, blood flow), and ICC integrity. *In vitro*, cell viability, apoptosis, mitochondrial membrane potential (MMP), and key proteins in the PINK1/Parkin mitophagy pathway were analyzed using CCK-8, flow cytometry, fluorescent probes, and Western blot.

**Results:**

IGFBP2 knockdown significantly ameliorated hyperglycemia, promoted gastric emptying, enhanced contractile activity, and improved gastric perfusion. Mechanistically, it enhanced ICC survival, suppressed apoptosis, reduced mitochondrial oxidative stress, and stabilized MMP. Crucially, these benefits were mediated by the downregulation of VDAC1 and subsequent inhibition of PINK1/Parkin-dependent mitophagy.

**Discussion:**

These findings indicate that IGFBP2 drives mitochondrial dysfunction and ICC loss *via* a VDAC1-dependent mitophagy pathway, revealing a previously underrecognized mechanism in DGP. The observed functional recovery and cytoprotection following IGFBP2 knockdown suggest that targeting this pathway may offer a potential therapeutic strategy for DGP.

**Conclusion:**

IGFBP2 acts as a potential upstream regulator that drives mitochondrial dysfunction and ICC loss in DGP *via* a VDAC1-mitophagy axis. These findings unveil IGFBP2 as a promising therapeutic target for intervening in the progression of DGP.

## 1 INTRODUCTION

Diabetic gastroparesis (DGP), characterized by delayed gastric emptying in the absence of mechanical obstruction, represents a prevalent gastrointestinal complication of diabetes mellitus [1]. The global diabetes epidemic underpins its rising burden, with projections indicating an increase to 783 million people with diabetes mellitus by 2045 [2]. Despite this trajectory, DGP remains underdiagnosed due to the non-specificity of its clinical manifestations, including nausea, vomiting, early satiety, postprandial fullness, and abdominal discomfort, which often mimic common functional dyspepsia (FD) [3]. Current diagnostic criteria require gastric emptying scintigraphy within 3 months of symptom onset, coupled with persistent symptoms >3 months [4], yet real-world evidence suggests significant under-identification given the complexity of longitudinal symptom assessment. Therapeutic options are equally constrained, as metoclopramide stands as the sole FDA-approved pharmacotherapy [5]. These diagnostic and therapeutic shortages stem directly from the elusive pathogenesis of DGP, which impedes targeted drug development and underscores the imperative to elucidate the underlying mechanisms.

DGP arises from a complex interplay of factors, including autonomic neuropathy, enteric nervous system dysfunction, and loss of the specialized pacemaker cells, the interstitial cells of Cajal (ICC) [6, 7]. ICC are essential for gastrointestinal motility, functioning as pivotal pacemakers that orchestrate gastric rhythmicity and serve as neuromodulatory intermediaries that critically regulate gastric emptying to maintain digestive homeostasis [8-10]. Alterations in ICC density, distribution, or ultrastructure are implicated in a spectrum of motility pathologies, including idiopathic achalasia, diabetic gastropathy, chronic intestinal pseudo-obstruction, constipation, and FD [11-13]. In the context of diabetes, hyperglycemia-induced oxidative stress and advanced glycation end products are known to inflict damage upon ICC, leading to their depletion and dysfunction, which are hallmarks of DGP [7]. While the mechanistic link between ICC deficiencies and dysmotility remains incompletely defined, prior investigations identified hyperactivated autophagy as a key mediator of ICC depletion and phenotypic aberration [14]. Mitophagy is a selective autophagic process that eliminates dysfunctional mitochondria following depolarization or oxidative insult, in which impaired organelles are sequestered within autophagosomes and delivered to lysosomes for degradation [15]. As reported, gastric dysmotility in an FD rat model was associated with marked autophagic hyperactivation, pronounced mitochondrial swelling, and prominent vacuolar degeneration within ICC [16]. Mitochondria are crucial for the high-energy demands of ICC, and their dysfunction, often precipitated by diabetic hyperglycemia, is increasingly recognized as a central event in the pathogenesis of DGP [7, 17]. Consequently, modulation of ICC mitophagy emerges as a plausible therapeutic strategy for restoring GI motor function.

Insulin-like growth factor binding proteins (IGFBPs) represent a family of secreted proteins that bind IGF ligands with high affinity. This interaction critically regulates the circulatory stability, bioavailability, and tissue-specific localization of IGFs [18]. Emerging evidence indicates that IGFBP2 is closely associated with diabetic nephropathy [19]. Notably, a recent study revealed that IGFBP2 promotes mitochondrial damage-induced podocyte apoptosis through the integrin α5/FAK signaling pathway in diabetic kidney disease [20], establishing a link between IGFBP2 and diabetes-related mitophagy. In the DGP transcriptomic dataset GSE130672, we observed a significant upregulation of IGFBP2 expression. According to StarBase analysis (https://starbase.sysu.edu.cn/, retrieved April 21, 2025), IGFBP2 can interact with voltage-dependent anion channel 1 (VDAC1), a mitochondrial membrane protein involved in mitophagy [21]. VDAC1, located on the outer mitochondrial membrane, acts as a critical gatekeeper for cellular metabolism and a key regulator of mitochondrial dysfunction. Its involvement in the formation of the mitochondrial permeability transition pore and its role in mediating mitophagy have been documented in various diabetic complications, including diabetic cardiomyopathy and nephropathy. Pharmacological or genetic suppression of VDAC1 has been shown to mitigate hyperglycemia-induced mitochondrial dysfunction in endothelial and fibroblast cell cultures, highlighting its potential as a therapeutic target [21, 22]. However, the role of IGFBP2 in DGP and its functional relationship with mitophagy remain unclear.

Therefore, this study aims to investigate the role of IGFBP2 in the pathogenesis of diabetic gastroparesis, with a specific focus on its impact on mitochondrial autophagy in ICC. We validated the regulatory relationship between IGFBP2 and VDAC1-mediated mitophagy through *in vitro* and *in vivo* models, and explored whether targeted inhibition of IGFBP2 could restore ICC integrity and gastric motility by ameliorating mitochondrial dysfunction. Our findings provide novel insights into the mechanisms underlying DGP and identify potential therapeutic targets for mitigating diabetic gastrointestinal complications.

## 2 MATERIALS AND METHODS

### 2.1 Animals and Ethics Statement

A total of 32 female C57BL/6J mice (6 weeks old; weighing 14-16 g) were obtained from Hangzhou Medical College and maintained under specific pathogen-free (SPF) conditions at a room temperature of 22-24 °C, 50-60% humidity, and a 12/12-h light/dark cycle. The animals were housed in groups of two or three per cage with free access to standard chow and water. All experimental protocols were conducted in accordance with the guidelines of the China Council on Animal Care and Use and were approved by the Institutional Animal Care and Use Committee (IACUC), Zhejiang Center of Laboratory Animals (Approval No. ZJCLA-IACUC-20011182).

### 2.2 Diabetic Model Construction

Diabetes was induced in mice *via* intraperitoneal administration of streptozotocin (STZ; 160 mg/kg; HY-13753, MedChemExpress, Monmouth Junction, NJ, USA) at 11:00 AM. Blood glucose monitoring was performed weekly at 3:00 PM until diabetes onset, after which measurements were taken every 48 h at the same time. Diabetes was defined as blood glucose levels exceeding 250 mg/dL on two consecutive tests. Animals failing to develop hyperglycemia after one week received up to two additional STZ injections at weekly intervals. Mice not exhibiting diabetic status following the third injection were excluded from the study. Blood samples were collected from the mandibular vascular bundle with strict volume control to prevent anemia. A subtherapeutic regimen of insulin glargine (Lantus^®^; Sanofi-Aventis US LLC, Paris, France) was administered daily *via* intraperitoneal injection when glucose levels surpassed 600 mg/dL to maintain survival while permitting sustained hyperglycemia (400-600 mg/dL) to facilitate the development of diabetic complications. Additionally, a vehicle control group (n = 8) received an equivalent volume of citrate buffer solution using the same injection protocol.

### 2.3 Adeno-associated Virus (AAV) Vector Administration

Recombinant AAV vectors AAV-shIGFBP2 (261973) and AAV-shRNA (7014) were obtained from Vector Biolabs (Malvern, PA, USA). Diabetic mice were randomly allocated into three experimental cohorts (n = 8 per group) and received intravenous injections of 0.9% sodium chloride, AAV-shRNA, or AAV-shIGFBP2, respectively. Diabetic mice received intraperitoneal injections of AAV resuspended in 200 µL of 0.9% sodium chloride at doses ranging from 1×10^12^ vector genomes (vg) per mouse.

### 2.4 Enzyme-linked Immunosorbent Assay (ELISA)

Serum concentrations of glycated hemoglobin (ELMM1098) were quantified using a commercial enzyme-linked immunosorbent assay (ELISA) kit (Lycor, Shanghai, China). Briefly, 50 µL of each serum sample and standard were dispensed in duplicate into microplate wells along with 50 µL of biotin-conjugated detection antibody, and the mixture was incubated for 2 h at room temperature. After washing, 100 µL of streptavidin-horseradish peroxidase (HRP) solution was introduced and incubated for 1 h. Chromogenic development was initiated by adding 100 µL of tetramethylbenzidine (TMB) substrate for 10 min and halted using 100 µL of stop solution. Absorbance was measured immediately at 450 nm using a Multiskan™ FC microplate reader (Thermo Scientific, Waltham, MA, USA).

### 2.5 Gastric Emptying

Each mouse was gavaged with 2 mL of 5% phenol red solution (PRL01, AmyJet Scientific, Wuhan, China). After 30 min, the animals were euthanized, and the stomach was excised and placed in a culture dish. Gastric tissues were rinsed thoroughly with distilled water, then treated with 1 mol/L NaOH and homogenized. Following 1 h of incubation, proteins were precipitated using 20% trichloroacetic acid for 30 min. The samples were centrifuged at 5,000 × g for 10 min at 4°C to isolate the supernatant containing phenol red. Absorbance of the supernatant was measured spectrophotometrically at 560 nm. Gastric emptying rate was calculated using the formula: Gastric emptying (%) = [1 − (phenol red recovered/standard phenol red volume)] × 100.

### 2.6 Gastric Peristalsis Analysis

Mice were anesthetized by inhaling isoflurane. After midline laparotomy, the stomach was carefully exposed. The pyloric sphincter was clipped, and gastric motor activity was recorded using a force transducer (Starm, Shanghai, China). Peristaltic frequency was calculated from a 10-min continuous recording, and the mean contraction amplitude was determined based on three representative motility cycles.

### 2.7 Gastric Blood Flow

A laser Doppler flowmetry probe (YUYAN INSTRUMENT, Shanghai, China) was gently applied to the gastric surface. Once a stable signal was achieved, blood flow was recorded over a 30-second period. The mean perfusion value obtained between 10 and 25 seconds was defined as the gastric blood flow for each animal.

### 2.8 Isolation, Culture, and Identification of ICC

ICC were isolated from mice euthanized under anesthesia by 100 mg/kg sodium pentobarbital (intravenous injection; P3761, Haoran Biological Technology, Shanghai, China). The stomach was aseptically excised, rinsed in phosphate-buffered saline (PBS, abs962, Absin, Shanghai, China), and minced. Tissue fragments were digested in 0.25% trypsin (C0201, Beyotime, Shanghai, China) at 37°C for 45 minutes. The resulting suspension was filtered and centrifuged at 21,000 × g for 5 min at 20°C. After supernatant removal, the pellet was washed in PBS and recentrifuged under the same conditions. ICC were subsequently cultured in L-DMEM (11885092, Thermo Scientific) supplemented with 10% FBS (26140079, Thermo Scientific) at 37°C in a 5% CO_2_ atmosphere. Western blot analysis was performed to detect the expression of c-kit (CD117), which is widely recognized as the most specific surface marker for ICC [23].

### 2.9 Cell Counting Kit-8 (CCK-8) Assay

ICC were plated in 96-well plates at a density of 5×10^3^ cells per well. Cell viability was assessed using the Cell Counting Kit-8 (ab228554, Abcam, Cambridge, UK) by adding 10 µL of WST-8 solution to each well, followed by incubation at 37 °C for 4 h in the dark. Absorbance was measured at 460 nm using a multiwell plate imager (CytoSMART Omni, Axion BioSystems, Shanghai, China).

### 2.10 Cell Apoptosis Assay

Cell apoptosis was assessed using an Annexin V-FITC/PI double-staining kit (C1062L, Beyotime) according to the manufacturer's protocol. Briefly, 1×10^5^ ICC were resuspended in 195 µL of binding buffer and incubated with 5 µL Annexin V-FITC and 10 µL propidium iodide (PI) for 15 min at room temperature in the dark. Samples were analyzed immediately using an Attune CytPix flow cytometer (Thermo Scientific) with 488 nm excitation. Data analysis was performed with the instrument’s dedicated software.

### 2.11 Mitochondrial Membrane Potential (MMP)

MMP was assessed using the JC-1 assay kit (C2003S, Beyotime). Briefly, ICC were seeded at 2×10^6^ cells/mL and then harvested and incubated with JC-1 staining solution for 20 min at 37 °C. After washing, cells were resuspended in assay buffer. Fluorescence was measured immediately using an Attune NxT flow cytometer.

MMP was also assessed using Mito-Tracker Red CMXRos (C1035, Beyotime). Cells were incubated with the Mito-Tracker working solution at 37 °C for 30 min. Following incubation, the nuclei were counterstained with 2-(4-Amidinophenyl)-6-indolecarbamidine dihydrochloride (DAPI). The probe solution was then carefully removed and replaced with fresh culture medium. Fluorescent images were acquired using a TCS SP8 laser scanning confocal microscope (Leica, Wetzlar, Germany).

### 2.12 Western Blot

Total protein or mitochondrial protein was extracted from gastric tissues or ICC with RIPA lysis buffer (R0010, Solarbio, Beijing, China)/Mitochondrial Protein Extraction Kit (EX1320, Solarbio) and quantified using a BCA assay (PC0020, Solarbio). Equal amounts of protein were denatured (95°C, 5 min), separated by electrophoresis on NuPAGE™ 4–12% Bis-Tris gels (NP0321BOX, Thermo Scientific), and transferred to PVDF membranes (YA1701, Solarbio). After 1 h of blocking at room temperature (37581, Thermo Scientific), the membranes were incubated overnight at 4 °C with the following primary antibodies: anti-IGFBP2 (ab188200, 1:1000, Abcam), anti-VDAC1 (ab186321, 1:1000, Abcam), anti-PINK1 (23274-1-AP, 1:1000, Proteintech, Wuhan, China), anti-Parkin (2132, 1:1000, CST, Beverly, MA, USA), anti-TOMM20 (ab186735, 1:1000, Abcam), anti-c-kit (ab317843, 1:1000, Abcam) and anti-β-actin (ab8226, 1:10,000, Abcam). HRP-conjugated anti-rabbit (ab175781, 1:10,000, Abcam) or anti-mouse (ab205719, 1:10,000, Abcam) secondary antibodies were incubated with the membranes for 1 h at room temperature. Protein bands were visualized using BeyoECL Plus substrate (P0018S, Beyotime) on an iBright CL750 imaging system (Thermo Scientific), and band intensity was quantified with ImageJ software (NIH, v1.52s) normalized to β-actin or TOMM20.

### 2.13 Quantitative Real-time PCR (qRT-PCR)

Total RNA was extracted from the ICC (4×10^5^ cells/ mL) with Trizol reagent (15596026, Invitrogen, USA). Reverse transcription was carried out using a reverse transcription kit (4368813, Applied Biosystems, USA) to synthesize cDNA from the total RNA. A qPCR reaction system was prepared according to the manufacturer’s instructions, with a total reaction volume of 20 μL. qPCR was performed using SYBR-Green PCR Master Mix (4364344, Applied Biosystems, USA) and the ABI Prism 7900 sequence detection system (Applied Biosystems; Thermo Fisher Scientific, Inc.). The specific primers (Sangon Biotech, China): IGFBP2 (forward: 5'-CAGACGCTACGCTGCTATCC-3', reverse: 5'-CCCTCAGAGTGGTCGTCATCA-3'), VDAC1 (forward: 5'-CCCACATACGCCGATCTTGG-3', reverse: 5'-GCTGCCGTTCACTTTGGTG-3'), and β-actin (forward: 5'-GGCTGTATTCCCCTCCATCG-3', reverse: 5'-CCAGTTGGTAACAATGCCATGT-3').

### 2.14 Statistical Analyses

Data are presented as mean ± SD from three independent experiments. Statistical analyses were performed using GraphPad Prism v8.0. Multiple groups were compared by one-way ANOVA or two-way ANOVA, with *p* < 0.05 considered significant.

## 3 RESULTS

### 3.1 IGFBP2 Knockdown Alleviates Systemic Hyperglycemia and Gastroparesis Phenotypes

To investigate the role of IGFBP2 in DGP, we established an *in vivo* model with stable knockdown of IGFBP2. Blood glucose levels were significantly lower in the IGFBP2 knockdown group compared to the control diabetic group (*p* < 0.001, Fig. **1A**). This improvement in glycemic control was further corroborated by a substantial reduction in the levels of glycated hemoglobin (HbA1c), a key indicator of long-term glucose homeostasis (*p* < 0.001, Fig. **1B**).

We next assessed the direct impact on gastric motor function. Gastric emptying, which is severely delayed in DGP, was significantly accelerated in mice with IGFBP2 deficiency (*p* < 0.001, Fig. **1C**). Furthermore, detailed *ex vivo* analysis of gastric smooth muscle activity revealed a pronounced enhancement in contractile function. Both the frequency and amplitude of spontaneous gastric contractions were significantly increased following IGFBP2 knockdown (*p* < 0.001, Fig. **1D**-**E**), indicating a restoration of coordinated gastric motility.

To explore a potential mechanism underlying the improved motor function, we measured gastric mucosal blood flow, as microvascular dysfunction is a known contributor to DGP. Laser Doppler flowmetry demonstrated that IGFBP2 silencing led to a significant increase in gastric blood flow (*p* < 0.01, Fig. **1F**), suggesting that improved tissue perfusion may be one mechanism through which IGFBP2 knockdown facilitates the recovery of gastric function.

### 3.2 IGFBP2 Knockdown Downregulates VDAC1 Expression and Enhances ICC Survival

To investigate the molecular mechanism by which IGFBP2 knockdown improves gastric function, we first assessed its impact on protein expression in gastric tissues. The expression of IGFBP2 and VDAC1 was increased in the DGP model group (*p* < 0.01; Fig. **1G**-**I**). Western blot analysis revealed that knockdown of IGFBP2 successfully and significantly reduced its protein levels compared to the shRNA group (*p* < 0.05; Fig. **1G** and **H**). Notably, this reduction in IGFBP2 was accompanied by a marked decrease in the expression of VDAC1, a pivotal mitochondrial outer membrane protein implicated in cellular apoptosis and metabolic dysfunction (*p* < 0.05, Fig. **1G**-**I**). This strong positive correlation suggests a potential regulatory relationship between IGFBP2 and VDAC1 in the context of diabetic gastroparesis.

Given the established role of VDAC1 in mediating cell death pathways, we next evaluated the functional consequences of IGFBP2 downregulation on ICC viability and apoptosis. We extracted and isolated ICC from mouse stomach tissue and identified them by Western blot analysis of c-kit protein expression. The results showed that ICC in the Vehicle group significantly expressed c-kit, whereas those in the DGP group exhibited a marked decrease in c-kit expression, and IGFBP2 knockdown restored c-kit expression (*p* < 0.001, Fig. **2A** and **B**). To confirm that AAV-mediated IGFBP2 knockdown successfully interfered with IGFBP2 expression at the cellular level, we performed qRT-PCR to assess IGFBP2 mRNA levels in ICC. The results showed that, compared with the shRNA group, IGFBP2 knockdown significantly reduced the expression of both IGFBP2 and VDAC1 mRNA in ICC (*p* < 0.001, Fig. **2C** and **D**).

The CCK-8 assay demonstrated that silencing IGFBP2 significantly enhanced the viability of ICC from diabetic mice (*p* < 0.001, Fig. **2C**), indicating a potent cytoprotective effect. Flow cytometric analysis using Annexin V-FITC/PI double staining showed that the percentage of apoptotic cells was substantially lower in the IGFBP2-knockdown group than in the shRNA group (*p* < 0.001, Fig. **2F** and **G**). These findings collectively indicate that IGFBP2 deficiency attenuates ICC apoptosis and promotes survival, effects that are potentially mediated through the downregulation of VDAC1.

### 3.3 IGFBP2 Knockdown Attenuates Mitochondrial Oxidative Stress and Stabilizes Membrane Potential

To investigate the protective effects of IGFBP2 deficiency on mitochondrial function, we assessed key indicators of oxidative stress and mitochondrial integrity. Mitochondrial superoxide production, as measured by MitoSOX Red fluorescence, was markedly reduced in ICC following IGFBP2 knockdown compared to the shRNA group (*p* < 0.001, Fig. **2H** and **I**), indicating a significant attenuation of oxidative stress.

Concomitantly, evaluation of mitochondrial membrane potential (MMP) using JC-1 staining revealed a profound stabilization of MMP in IGFBP2-deficient cells. Flow cytometric analysis demonstrated a significantly increased JC-1 aggregate/monomer ratio, reflecting enhanced mitochondrial polarization and improved energetic capacity (*p* < 0.001, Fig. **3A** and **B**).

### 3.4 IGFBP2 Knockdown Inhibits Mitophagy Activation in ICC

To investigate the molecular mechanism by which IGFBP2 deficiency preserves mitochondrial function, we assessed the activation of the mitophagy pathway, a key quality control process that is dysregulated under diabetic conditions. Western blot analysis revealed that knockdown of IGFBP2 significantly suppressed the mitophagy cascade in ICC.

As depicted in Fig. (**3C**-**E**), the protein expression of key mitophagy initiators was markedly altered. The stabilization of PINK1 on the outer mitochondrial membrane and the subsequent recruitment of the E3 ubiquitin ligase Parkin, which are hallmarks of mitophagy induction, were both profoundly decreased upon IGFBP2 silencing (*p* < 0.05). These results demonstrate that IGFBP2 knockdown exerts a protective effect by inhibiting the hyperactivation of the PINK1/Parkin-mediated mitophagy pathway, thereby preserving mitochondrial integrity and cellular homeostasis.

## 4 DISCUSSION

Our study unveils a novel and critical role of IGFBP2 in the pathogenesis of DGP, with a specific focus on the ICC. The principal finding is that knockdown of IGFBP2 confers comprehensive protective effects, markedly ameliorating systemic hyperglycemia and restoring gastric motility in a DGP model. This multifaceted improvement is demonstrated by a series of key observations: the alleviation of core diabetic phenotypes (reduced blood glucose and HbA1c levels) and the normalization of central gastric functions (accelerated gastric emptying, enhanced contractile frequency and amplitude, and improved gastric blood flow). Critically, we identified that the cellular basis for this recovery lies within the ICC, where IGFBP2 deficiency preserved cellular viability, suppressed apoptosis, and was intimately linked to the amelioration of mitochondrial dysfunction and the inhibition of aberrant mitophagy activation through the downregulation of VDAC1. Collectively, these results strongly suggest that IGFBP2 is not merely a bystander but an active pathogenic driver in DGP, primarily by disrupting the integrity and function of the pivotal ICC.

The pathogenesis of DGP is multifactorial, involving intricate interactions among metabolic, neural, immune, and hormonal factors. A central abnormality is the depletion and ultrastructural damage of ICC, which serve as gastric pacemakers. This is largely attributed to hyperglycemia-induced oxidative stress and reduced insulin/IGF-1 signaling, leading to diminished stem cell factor (SCF) production from smooth muscle cells [24, 25]. Concurrently, an imbalance in macrophage phenotypes—characterized by a predominance of pro-inflammatory M1 cells over cytoprotective M2 macrophages—promotes cytokine-mediated injury to both ICC and enteric neurons [24, 25]. Enteric neuropathy, particularly the impairment of nitrergic neurons, further disrupts pyloric relaxation and gastric accommodation [7, 26]. These structural and functional deficits are modulated by dysregulation of the brain-gut axis, including altered secretion of ghrelin and motilin, while chronic hyperglycemia itself serves as a key exacerbating factor [1, 26]. Clinically, the poor correlation between symptom severity and gastric emptying rates underscores the necessity of objective diagnostic tools such as scintigraphy [26]. Management follows a stepwise approach, incorporating dietary adjustments, glycemic optimization, and prokinetic agents like metoclopramide, though its use is constrained by neurological adverse effects [7, 26]. For patients refractory to pharmacotherapy, gastric electrical stimulation and G-POEM may provide symptomatic relief, albeit with careful patient selection [1, 26]. In summary, DGP arises from integrated cellular and immune disturbances.

IGFBP2 has emerged as a promising biomarker for diabetic kidney disease (DKD) and related clinical outcomes [27]. Through multi-omics analysis, IGFBP2, along with B2M and CST3, was identified as a validated serum panel showing stage-dependent elevation during DKD progression [28]. In a cohort of 436 individuals with type 2 diabetes followed over 8 years, higher baseline plasma IGFBP2 was an independent predictor of subsequent eGFR decline [27]. Furthermore, elevated IGFBP2 levels were associated with increased risks of both all-cause and cardiovascular mortality in large type 2 diabetes populations, with one study confirming this association was independent of metformin use and insulin sensitivity [27, 29]. Collectively, these findings support IGFBP2 as a non-invasive marker for renal function deterioration and adverse prognosis in diabetes.

The precise molecular pathway through which IGFBP2 ablation preserves ICC integrity appears to involve VDAC1, a critical gatekeeper of mitochondrial homeostasis. Our data demonstrated that IGFBP2 knockdown concomitantly reduced both IGFBP2 and VDAC1 protein levels in gastric tissues, suggesting a potential regulatory axis. This finding aligns with evidence that VDAC1, beyond its role in metabolite transport, is a central node in mitochondria-mediated apoptosis and mitophagy [20, 30]. Specifically, VDAC1 oligomerization forms a conduit for cytochrome c release, precipitating caspase activation, and serves as a platform for mitophagy receptors such as Parkin [31, 32].

The observed downregulation of VDAC1 following IGFBP2 silencing likely underlies the amelioration of mitochondrial dysfunction, evidenced by restored membrane potential, dampened oxidative stress, and suppressed apoptosis in ICCs. Based on the established role of IGFBP2 as a transcriptional regulator, as demonstrated by its control of the CD24 promoter in glioblastoma cells [33]. It is plausible that it may similarly govern the expression of VDAC1. The bioinformatic prediction from StarBase provides direct support for a potential regulatory relationship between IGFBP2 and VDAC1. Therefore, we hypothesize that IGFBP2 upregulation might enhance VDAC1 transcription, which could subsequently lead to mitochondrial dysfunction and facilitate apoptosis in ICC.

The pathological role of dysregulated mitochondrial quality control, particularly excessive mitophagy, in DGP is strongly supported by our findings. We demonstrate that IGFBP2 knockdown suppresses hyperactivation of the PINK1/Parkin-mediated mitophagy pathway, thereby preserving mitochondrial function and ICC viability. This aligns with studies in functional dyspepsia models, where inhibiting excessive mitophagy restored ICC networks and motility [34-36].

Although electroacupuncture was shown to ameliorate DGP by promoting autophagy [37], this apparent contradiction highlights the dual role of autophagy: restoring beneficial baseline clearance *versus* inhibiting pathological overactivation [15, 38]. Thus, therapeutic strategies must precisely modulate mitophagic flux based on disease context.

Collectively, this study identifies IGFBP2 as a potential upstream modulator associated with VDAC1-PINK1/Parkin-mediated mitophagy in ICCs, providing preliminary evidence for a mechanistic link between metabolic stress and gastric motility failure in DGP. The concurrent improvement in glycemic control and gastric motility upon IGFBP2 knockdown supports the exploration of IGFBP2 as a potential therapeutic target in DGP management. Our findings suggest that targeted inhibition of IGFBP2, could preserve mitochondrial integrity and prevent ICC loss, offering a fundamentally new strategy beyond symptomatic management.

However, this study has limitations. While we demonstrate a strong correlation, the causal necessity of the IGFBP2-VDAC1 axis requires validation through rescue experiments (*e.g.*, VDAC1 overexpression). Furthermore, the findings from animal models need verification in human tissues, and the upstream signals causing IGFBP2 upregulation in diabetes remain unexplored.

Building upon these findings and limitations, future work will focus on definitively establishing the causal relationship through mechanistic rescue studies. The therapeutic potential of this pathway will be further explored by testing specific IGFBP2 inhibitors in more physiologically complex models, such as aged diabetic animals. Concurrently, efforts will be made to validate the dysregulation of this pathway in human patient-derived tissues or organoids to assess its clinical relevance.

## CONCLUSION

This study clarifies the regulatory role of IGFBP2 in DGP. Elevated IGFBP2 accelerates ICC damage and mitochondrial dysfunction by activating VDAC1-related PINK1/Parkin mitophagy signaling. Silencing IGFBP2 can relieve hyperglycemia, restore gastric motor function, improve mitochondrial homeostasis, and maintain ICC survival. IGFBP2 serves as a functional mediator in DGP progression, and targeting IGFBP2 may represent a potential therapeutic strategy for DGP.

## Figures and Tables

**Fig. (1):**
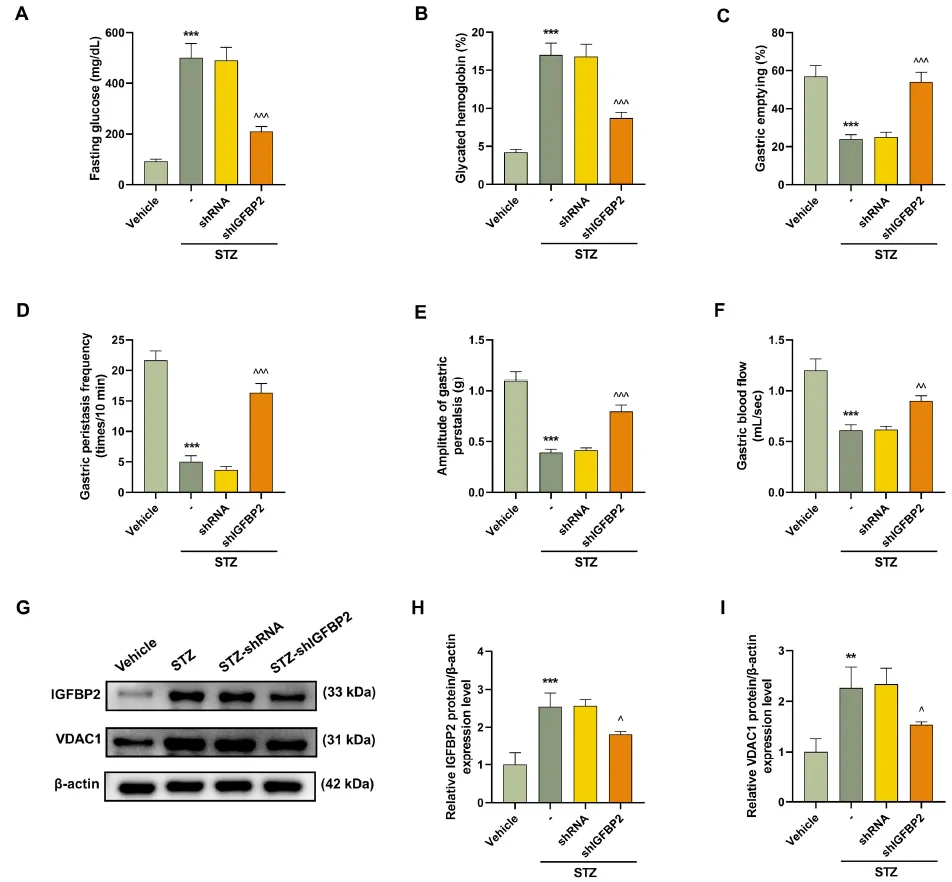
IGFBP2 knockdown improves glycemic control and gastric motility in a DGP model. (**A**) Blood glucose levels measured by a glucometer. (**B**) Glycated hemoglobin (HbA1c) levels detected by ELISA. (**C**) Gastric emptying assessed by phenol red assay. (**D** and **E**) Gastric motility frequency and amplitude were recorded *via* a force transducer. (**F**) Gastric blood flow measured by laser Doppler flowmetry. (**G** - **I**) Representative Western blots and quantification of IGFBP2 and VDAC1 protein expression in gastric tissues. **p < 0.01, ***p < 0.001 *vs*. Vehicle; ^^^*p* < 0.05, ^^^^*p* < 0.01, ^^^^^*p* < 0.001 *vs*. shRNA. IGFBP2, Insulin-like Growth Factor Binding Protein 2; DGP, Diabetic gastroparesis; ELISA, Enzyme-linked immunosorbent assay; VDAC1, voltage-dependent anion channel 1; ICC, Interstitial Cells of Cajal; STZ: streptozotocin.

**Fig. (2):**
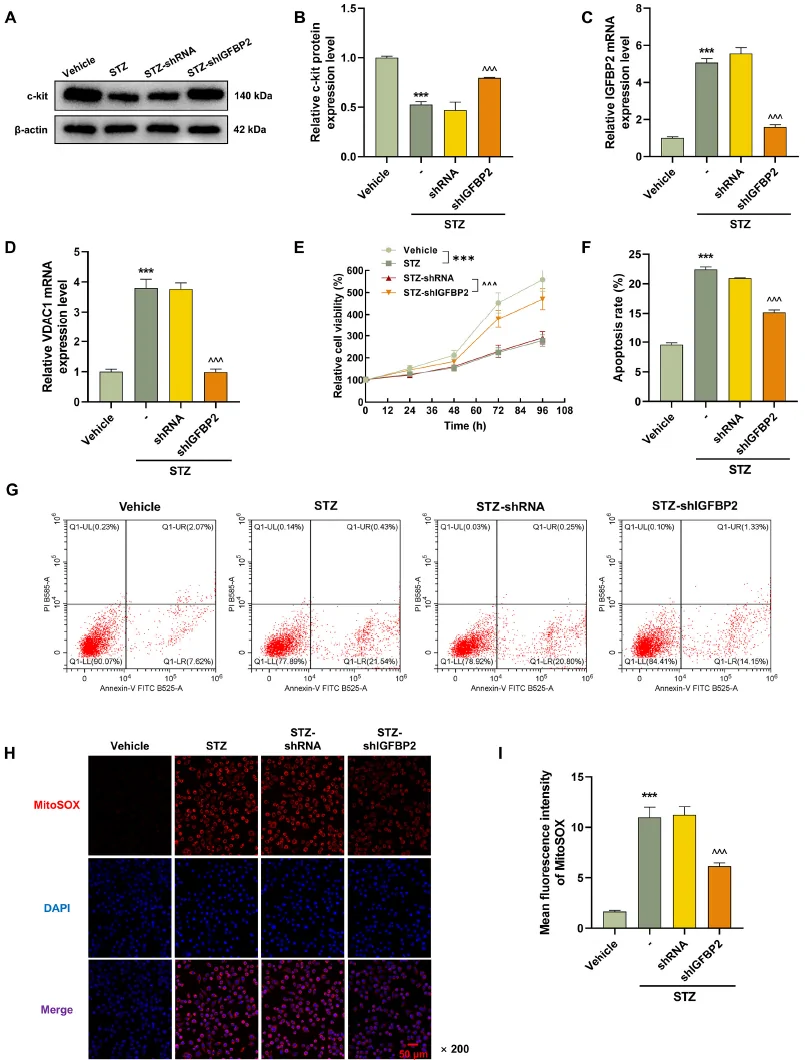
IGFBP2 knockdown attenuates mitochondrial oxidative stress and apoptosis in ICC. (**A** and **B**) Western blot analysis of c-kit protein expression. (**C** and **D**) Analysis of IGFBP2 and VDAC1 Expression in ICC Using qRT-PCR. (**E**) Cell viability of ICC assessed by CCK-8 assay. (**F** and **G**) Apoptosis rate evaluated by flow cytometry with Annexin V/PI staining. (**H**-**I**) MitoSOX Red staining measured mitochondrial superoxide production (×200, 50 µm). ***p < 0.001 *vs*. Vehicle; ^^^^^*p* < 0.001 *vs*. shRNA. CCK-8, Cell Counting Kit-8.

**Fig. (3):**
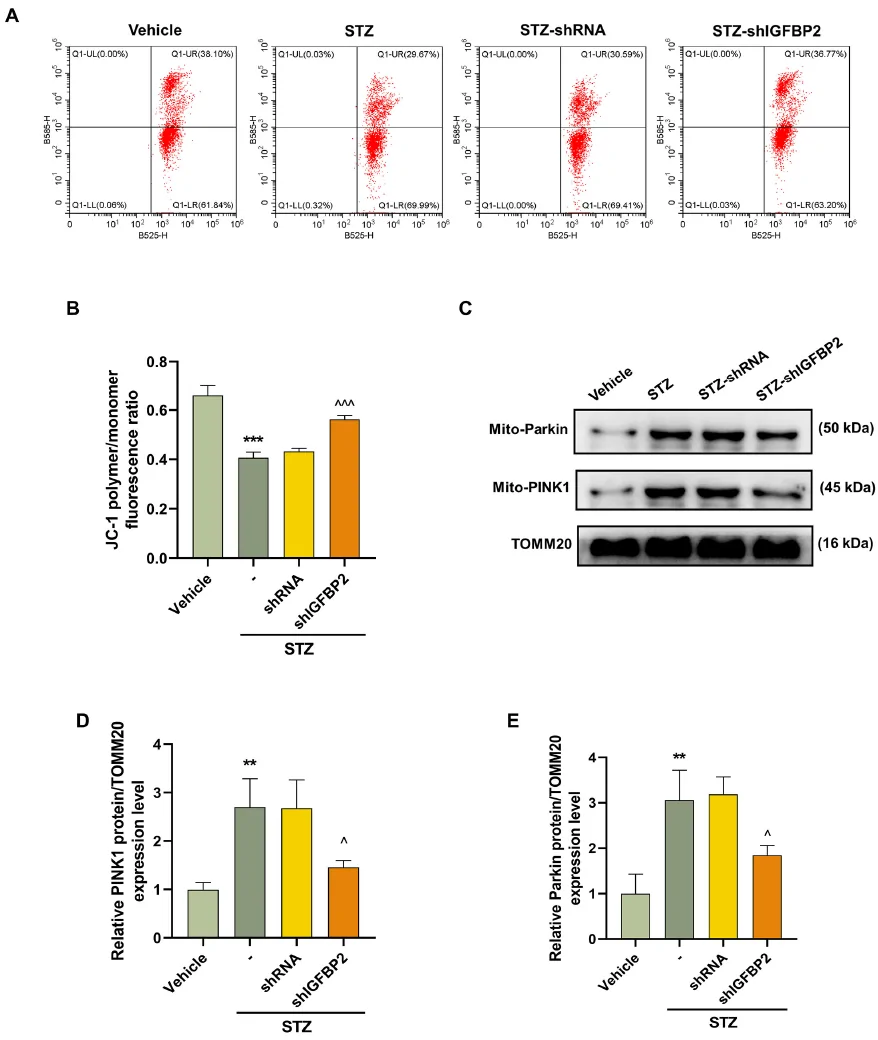
IGFBP2 knockdown stabilizes mitochondrial membrane potential and suppresses mitophagy. (**A** and **B**) Mitochondrial membrane potential assessed by JC-1 staining *via* flow cytometry. (**C** - **E**) Western blot analysis of mitophagy-related proteins (PINK1, Parkin). **p < 0.01, ***p < 0.001 *vs*. Vehicle; ^^^*p* < 0.05, ^^^^^*p* < 0.001 *vs*. shRNA.

## Data Availability

The data that support the findings of this study are available from the corresponding author, HZ, on special request.

## LIST OF ABBREVIATIONS

AAV: Adeno-associated Virus
ANOVA: Analysis of Variance
B2M: Beta-2-microglobulin
BCA: Bicinchoninic Acid
CCK-8: Cell Counting Kit-8
cDNA: Complementary DNA
CST3: Cystatin C
DGP: Diabetic Gastroparesis
DKD: Diabetic Kidney Disease
DMEM: Dulbecco's Modified Eagle Medium
eGFR: Estimated Glomerular Filtration Rate
ELISA: Enzyme-linked Immunosorbent Assay
FBS: Fetal Bovine Serum
FD: Functional Dyspepsia
FITC: Fluorescein Isothiocyanate
G-POEM: Gastric Peroral Endoscopic Myotomy
HbA1c: Glycated Hemoglobin
HRP: Horseradish Peroxidase
IACUC: Institutional Animal Care and Use Committee
ICC: Interstitial Cells of Cajal
IGF: Insulin-like Growth Factor
IGFBP2: Insulin-like Growth Factor Binding Protein 2
L-DMEM: Low-glucose Dulbecco's Modified Eagle Medium
MMP: Mitochondrial Membrane Potential
PBS: Phosphate-Buffered Saline
PI: Propidium Iodide
PINK1: PTEN-Induced Putative Kinase 1
PVDF: Polyvinylidene Difluoride
qRT-PCR: Quantitative Real-time Polymerase Chain Reaction
SCF: Stem Cell Factor
SPF: Specific Pathogen-free
STZ: Streptozotocin
TMB: Tetramethylbenzidine
TOMM20: Translocase of Outer Mitochondrial Membrane 20
VDAC1: Voltage-Dependent Anion Channel 1
